# Bacterial Community Composition and Dynamics Spanning Five Years in Freshwater Bog Lakes

**DOI:** 10.1128/mSphere.00169-17

**Published:** 2017-06-28

**Authors:** Alexandra M. Linz, Benjamin C. Crary, Ashley Shade, Sarah Owens, Jack A. Gilbert, Rob Knight, Katherine D. McMahon

**Affiliations:** a Department of Bacteriology, University of Wisconsin—Madison, Madison, Wisconsin, USA; b Department of Microbiology and Molecular Genetics, Michigan State University, East Lansing, Michigan, USA; c Biosciences Division, Argonne National Laboratory, Argonne, Illinois, USA; d Computation Institute, University of Chicago, Chicago, Illinois, USA; e Department of Ecology and Evolution, Department of Surgery, University of Chicago, Chicago, Illinois, USA; f Center for Microbiome Innovation, Jacobs School of Engineering, University of California, San Diego, La Jolla, California, USA; g Department of Pediatrics, University of California, San Diego School of Medicine, La Jolla, California, USA; h Department of Computer Science and Engineering, Jacobs School of Engineering, University of California San Diego, La Jolla, California, USA; i Department of Civil and Environmental Engineering, University of Wisconsin—Madison, Madison, Wisconsin, USA; University of Minnesota

**Keywords:** 16S rRNA, freshwater, microbial communities, microbial ecology, time series

## Abstract

Bacteria play a key role in freshwater biogeochemical cycling, but long-term trends in freshwater bacterial community composition and dynamics are not yet well characterized. We used a multiyear time series of 16S rRNA gene amplicon sequencing data from eight bog lakes to census the freshwater bacterial community and observe annual and seasonal trends in abundance. The sites that we studied encompassed a range of water column mixing frequencies, which we hypothesized would be associated with trends in alpha and beta diversity. Each lake and layer contained a distinct bacterial community, with distinct levels of richness and indicator taxa that likely reflected the environmental conditions of each lake type sampled, including *Actinobacteria* in polymictic lakes (i.e., lakes with multiple mixing events per year), *Methylophilales* in dimictic lakes (lakes with two mixing events per year, usually in spring and fall), and “*Candidatus* Omnitrophica” in meromictic lakes (lakes with no recorded mixing events). The community present during each year at each site was also surprisingly unique. Despite unexpected interannual variability in community composition, we detected a core community of taxa found in all lakes and layers, including *Actinobacteria* tribe acI-B2 and *Betaprotobacteria* lineage PnecC. Although trends in abundance did not repeat annually, each freshwater lineage within the communities had a consistent lifestyle, defined by persistence, abundance, and variability. The results of our analysis emphasize the importance of long-term multisite observations, as analyzing only a single year of data or one lake would not have allowed us to describe the dynamics and composition of these freshwater bacterial communities to the extent presented here.

**IMPORTANCE** Lakes are excellent systems for investigating bacterial community dynamics because they have clear boundaries and strong environmental gradients. The results of our research demonstrate that bacterial community composition varies by year, a finding which likely applies to other ecosystems and has implications for study design and interpretation. Understanding the drivers and controls of bacterial communities on long time scales would improve both our knowledge of fundamental properties of bacterial communities and our ability to predict community states. In this specific ecosystem, bog lakes play a disproportionately large role in global carbon cycling, and the information presented here may ultimately help refine carbon budgets for these lakes. Finally, all data and code in this study are publicly available. We hope that this will serve as a resource for anyone seeking to answer their own microbial ecology questions using a multiyear time series.

## Abstract (precis)

Lakes are excellent systems for investigating bacterial community dynamics because they have clear boundaries and strong environmental gradients. The results of our research demonstrate that bacterial community composition varies by year, a finding which likely applies to other ecosystems and has implications for study design and interpretation. Understanding the drivers and controls of bacterial communities on long time scales would improve both our knowledge of fundamental properties of bacterial communities and our ability to predict community states. In this specific ecosystem, bog lakes play a disproportionately large role in global carbon cycling, and the information presented here may ultimately help refine carbon budgets for these lakes. Finally, all data and code in this study are publicly available. We hope that this will serve as a resource for anyone seeking to answer their own microbial ecology questions using a multiyear time series.

## INTRODUCTION

One of the major goals of microbial ecology is to predict bacterial community composition. However, we have only a superficial knowledge of the factors that would allow us to predict bacterial community dynamics. To characterize the diversity and dynamics of an ecosystem’s bacterial community, sampling the same site multiple times is just as necessary as sampling replicate ecosystems. Additionally, the sampling frequency must match the rate of change of the process being studied. We must first understand the scales on which bacterial communities change before we can design experiments that capture a full range of natural variation.

Bacterial communities have the potential to change more quickly than communities of macroorganisms due to their high rate of reproduction. A meta-analysis of a time series spanning 1 to 3 years found positive species-time relationships, indicating that more taxa were observed as the duration of sampling increased, due to incomplete sampling, extinction and immigration, or speciation (1). Bacterial time series display time decay, meaning that the community continues to become more dissimilar from that present at the initial sampling event as the time from that event increases (2). In one freshwater lake, the amount of change in the bacterial community over a single day was equivalent to the dissimilarity between the communities collected at sampling points 10 m apart (3). Conversely, bacterial communities can also change gradually over extremely long time scales, as they are sensitive to changes in environmental parameters such as nutrient availability and temperature. Wetland ecosystems and their carbon emissions are expected to change on scales of greater than 300 years (4); as these emissions are the result of bacterial processes, we expect that the bacterial community will change on the same time scale as its ecosystem. Changes in marine phytoplankton regimes have been observed to have occurred over the past millennium, correlating with shifts in climate (5). With such a large range of potential time scales for change, we now recognize the need to more rigorously consider the duration and frequency of sampling in microbial ecology.

Multiyear studies of bacterial communities are less common due to their logistical difficulties and the need for stable funding, but results from the United States National Science Foundation-funded Microbial Observatory and Long Term Ecological Research (LTER) projects are exemplary. As a few examples among many, the San Pedro North Pacific Microbial Observatory contributed to our understanding of heterogeneity of bacterial communities across space and time (6), while research at the Sapelo Island Microbial Observatory has led the field in linking genomic data to metadata (7). While there are several well-established long-term time series in marine systems, studies at this scale in freshwater are rare. In our own North Temperate Lakes Microbial Observatory, based in Wisconsin in the United States, a multiyear time series of metagenomic data was used to study sweeps in diversity at the genome level (8), adding to our knowledge of how genetic mutation influences bacterial communities. Long-term microbial ecology studies have a time-tested role in the quest to forecast bacterial communities.

Samples for our North Temperate Lakes Microbial Observatory time series were collected from eight bog lakes near Minocqua in the boreal region of northern Wisconsin in the United States. Bog lakes contain high levels of dissolved organic carbon in the form of humic and fulvic acids, resulting in dark, “tea-colored” water. Due to their dark color, bog lakes absorb heat from sunlight, resulting in strong stratification during the summer. The top layer in a stratified lake, called the “epilimnion,” is oxygen rich and warm. At the lake bottom, a cold layer called the “hypolimnion” is formed, becoming anoxic almost immediately in darkly stained bog lakes. The transitions between mixing of these two layers and stratification occur rapidly in these systems and at different frequencies (called mixing regimes) depending on the depth, surface area, and wind exposure of the lake. Changes in bacterial community composition along the vertical gradients established during stratification are well documented (9, 10). Mixing has been shown to represent a disturbance to the bacterial communities in bog lakes (11). The bacterial communities in bog lakes are still being characterized but contain both ubiquitous freshwater organisms (12, 13) and members of the candidate phylum radiation (14). Seasonality in freshwater lakes is thought to be the norm rather than the exception (15, 16); however, multiple years of sampling are needed to confirm these prior findings.

Our data set was comprised of 1,387 16S rRNA gene amplicon sequencing samples, collected from eight lakes and two thermal layers over 5 years. Our primary goals for this data set were to census members of the bog lake bacterial community and to identify taxa that are core to the bacterial community of bog lake ecosystems. We hypothesized that the mixing regime structures the bacterial community, leading to an association between mixing frequency and alpha and beta diversity in bog lakes. Finally, we investigated seasonality at the community level, clade level (roughly equivalent to the genus level), and operational taxonomic unit (OTU) level to identify annual trends. This extensive, long-term sampling effort establishes a time series that allows us to assess variability, responses to mixing frequency, and recurring trends in freshwater bacterial communities.

## RESULTS

### Overview of community composition.

We used a time series of 16S rRNA gene amplicon data to investigate bacterial community composition over time and across lakes. Sampling occurred at approximately weekly intervals and primarily during the summer stratified period (May to August) (see Fig. S1 in the supplemental material). Sites were not sampled continuously over the entire time series, and metadata are available for only a subset of samples. A total of 8,795 OTUs were detected in 1,387 samples. As is typical for most freshwater ecosystems, *Proteobacteria*, *Actinobacteria*, *Bacteroidetes*, and *Verrucomicrobia* were the most abundant phyla (Fig. S2). Within these phyla, levels of OTU abundance were highly uneven. Much of the abundance of *Proteobacteria* could be attributed to OTUs belonging to the well-known freshwater groups *Polynucleobacter* and *Limnohabitans*, and the freshwater lineage acI contributed disproportionately to the observed abundance of *Actinobacteria*. As is seen with many microbial communities, unevenness was a recurring theme in this data set, which had a long tail of rare OTUs and trends driven largely by the most abundant OTUs (17, 18). These results show that the composition of our data set is consistent with results from other bog lakes (10, 14).

10.1128/mSphere.00169-17.1 FIG S1  Sampling frequency and paired environmental data. Lakes were sampled during stratification each summer (June to August). Some sites include additional early spring samples (April and May) and fall samples (September to November). Not every lake was sampled in every year of sampling; every lake was sampled in 2007. Layer codes (third letter in the site designation on the *y* axis) of “U” indicate that the entire water column was sampled, rather than splitting samples by epilimnion and hypolimnion. Temperature and dissolved oxygen levels were measured throughout the water column during the collection of every sample in our data set. pH, DIC/DOC (dissolved inorganic carbon/dissolved organic carbon), TN/TDN (total nitrogen/dissolved nitrogen), and TP/TDP (total phosphorus/dissolved phosphorus) were not measured every year in every site. Instrumented buoys, maintained by North Temperate Lakes Long Term Ecological Research, are located on a subset of the sampling locations. Download FIG S1, EPS file, 2.3 MB. Copyright © 2017 Linz et al. 2017 Linz et al. This content is distributed under the terms of the Creative Commons Attribution 4.0 International license.

10.1128/mSphere.00169-17.2 FIG S2  Phylum rank abundance in entire data set. When OTUs are grouped by phylum and read abundances summed over the entire data set, *Proteobacteria*, *Actinobacteria*, and *Bacteroidetes* represent the most abundant phyla. Unclassified *Bacteria* represent the fifth largest group. Members of the candidate phylum radiation such as OD1 (“*Candidatus* Parcubacteria”) and OP3 (“*Candidatus* Omnitrophica”) are also well represented in this data set. Download FIG S2, EPS file, 1.5 MB. Copyright © 2017 Linz et al. 2017 Linz et al. This content is distributed under the terms of the Creative Commons Attribution 4.0 International license.

### Community richness.

**FIG 1:**
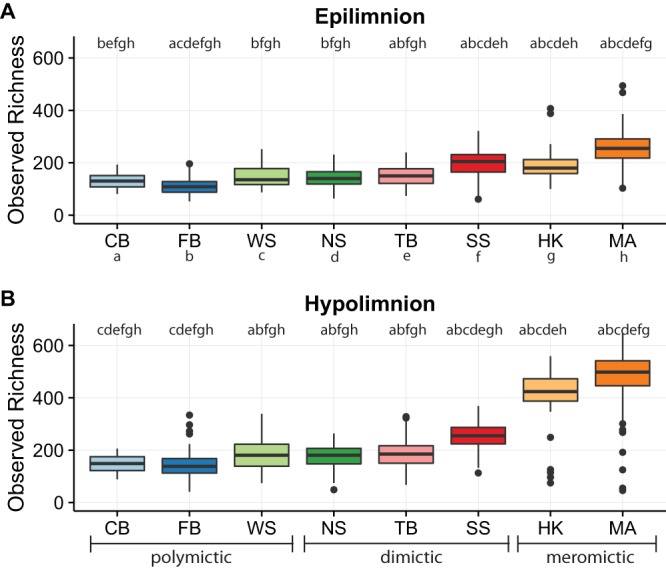
Richness by layer and lake. Lakes listed on the *x* axis are arranged by depth (see Table 1 for lake abbreviations and depth measurements). Significance (represented by letters for each lake above their box plot; the letters identifying each lake are below the *x* axis in panel A) was tested using a Wilcoxon signed-rank test with a Bonferroni correction for multiple pairwise comparisons, reported in Table S1. (A) Epilimnion. (B) Hypolimnion.

We hypothesized that water column mixing frequency was associated with alpha diversity. Observed richness was calculated for every sample at the OTU level, and samples were aggregated by lake and layer. Hypolimnia generally showed more richness than epilimnia (Fig. 1; see also Table S1 in the supplemental material). Significant differences in richness between lakes were detected using the Wilcoxon signed-rank test with a Bonferroni correction for multiple pairwise comparisons (Table S2). For both layers, polymictic lakes (i.e., lakes with multiple mixing events per year) had the fewest taxa, dimictic lakes (lakes with two mixing events per year, usually in spring and fall) had intermediate numbers of taxa, and meromictic lakes (lakes with no recorded mixing events) had the most taxa. This data set includes data from two fall mixing events (Trout Bog 2007 and North Sparkling Bog 2008), as well as from the artificial mixing event in North Sparkling Bog in 2008 (11). Richness decreased sharply in mixed samples compared to those taken during the summer stratified period (Fig. S3). The observed association between mixing frequency and richness suggests that water column mixing (or one or more covarying environmental parameters) structures the bacterial community.

10.1128/mSphere.00169-17.3 FIG S3  Richness over time during mixing events. In panels A and B, the black line traces the number of OTUs observed at each time point in the hypolimnion. Panels C and D show temperatures throughout the water column on each sampling date. Sharp decreases in richness are observed for both the fall mixing in Trout Bog, 2007 (A and C) and the artificial mixing in July in North Sparkling Bog, 2008 (B and D). Transient mixing dates in the fall of 2008 in North Sparkling Bog also show lower richness. Download FIG S3, EPS file, 2.6 MB. Copyright © 2017 Linz et al. 2017 Linz et al. This content is distributed under the terms of the Creative Commons Attribution 4.0 International license.

10.1128/mSphere.00169-17.8 TABLE S1  *P* values from comparison of richness between sites in Fig. 1. Observed levels of richness within layers were compared between lakes using a Wilcoxon signed-rank test with a Bonferroni correction for multiple pairwise comparisons. Download TABLE S1, DOCX file, 0.01 MB. Copyright © 2017 Linz et al. 2017 Linz et al. This content is distributed under the terms of the Creative Commons Attribution 4.0 International license.

**FIG 2:**
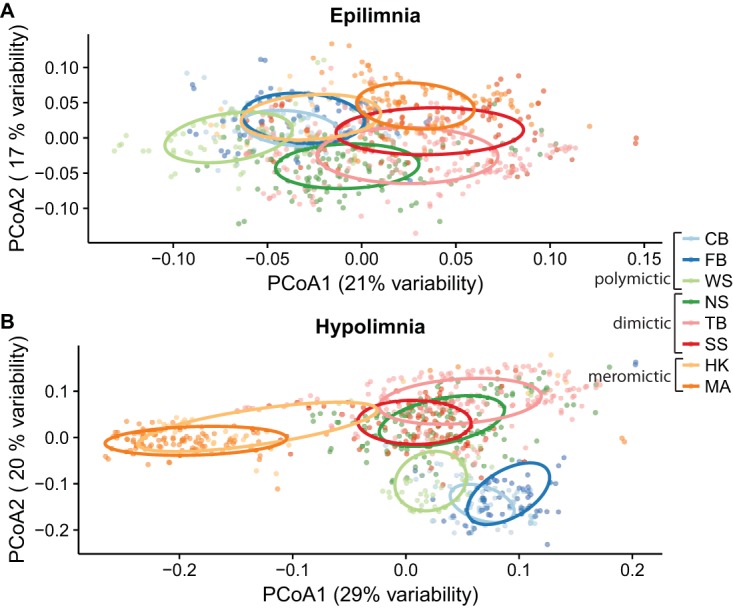
Principal-coordinate analysis of samples by layer. Weighted UniFrac distance values were used to perform principal-coordinate analysis on epilimnion (A) and hypolimnion (B) samples. The percentage of variance explained by the first two axes is reported in the axis labels. In both layers, samples cluster significantly by lake and mixing regime as tested using PERMANOVA (Table S2). (See Table 1 for lake abbreviations.) Data represented by ellipses indicating the clustering of each lake were calculated on the basis of standard errors using a 95% confidence interval. Differences in bacterial community composition between lakes and mixing regimes are more pronounced in hypolimnia than epilimnia. Additional plots of this ordination colored by other factors are shown in Fig. S5.

**FIG 3:**
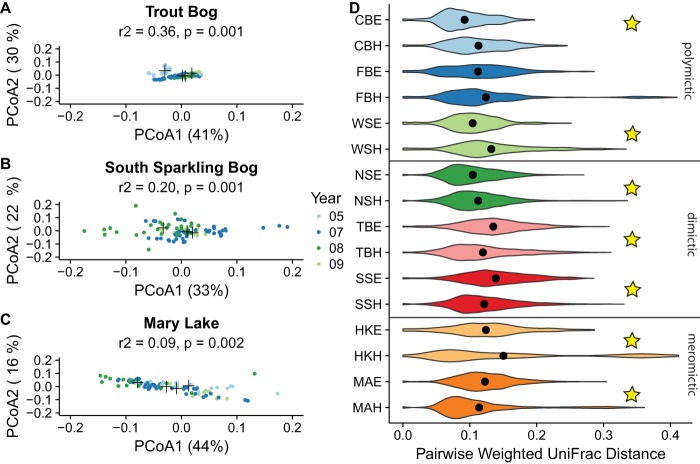
Interannual variability and dispersion by lake. (A to C) Principal-coordinate analysis using weighted UniFrac as the distance metric was used to measure the amount of interannual variation in the three lake hypolimnia with the longest time series (Trout Bog [A], South Sparkling Bog [B], and Mary Lake [C]). Additional ordinations of lake epilimnia are provided as supplemental figures (Fig. S5). Black crosses indicated the centroid for each year. All hypolimnia showed significant clustering by year by PERMANOVA (Table S2). Six outliers in Mary Lake from 2007 are not shown, as their coordinates lie outside the range specified for consistency between plots; these points were included in the PERMANOVA significance test. (D) Pairwise weighted UniFrac distance values within each lake and layer, including all samples. Stars indicate significant differences between layers at *P* = <0.05 by a Wilcoxon signed-rank test with a Bonferroni correction for multiple pairwise comparisons. 05, 2005; 07, 2007; 08, 2008; 09, 2009; CBE, Crystal Bog epilimnia; CBH, Crystal Bog hypolimnia; FBE, Forestry Bog epilimnia; FBH, Forestry Bog hypolimnia; WSE, West Sparkling Bog epilimnia; WSH, West Sparkling Bog hypolimnia; NSE, North Sparkling Bog epilimnia; NSH, North Sparkling Bog hypolimnia; TBE, Trout Bog epilimnia; TBH, Trout Bog hypolimnia; SSE, South Sparkling Bog epilimnia; SSH, South Sparkling Bog hypolimnia; HKE, Hell's Kitchen epilimnia; HKH, Hell's Kitchen hypolimnia; MAE, Mary Lake epilimnia; MAH, Mary Lake hypolimnia.

10.1128/mSphere.00169-17.9 TABLE S2  PERMANOVA tables. The results of PERMANOVA, implemented using Adonis() from the R package “vegan,” are shown here. The significant clustering by lake and mixing regime in the principal-coordinate analysis whose results are presented in Fig. 2 is supported, as is the clustering by year within lakes shown in Fig. 3A to C. Download TABLE S2, DOCX file, 0.01 MB. Copyright © 2017 Linz et al. 2017 Linz et al. This content is distributed under the terms of the Creative Commons Attribution 4.0 International license.

### Clusters of community composition.

To determine if mixing frequency is associated with community composition, we measured beta diversity between sites, based on the relative number of reads assigned to each OTU. When differences in community composition were quantified using weighted UniFrac distance and visualized using principal-coordinate analysis (PCoA), several trends emerged. The large number of samples precluded much interpretation using a single PCoA, but sample clustering by layer, mixing regime, and lake was evident. Thus, we also performed PCoA for single lakes (both layers). Communities from the epilimnion and hypolimnion layers were significantly distinct from each other at *P* = <0.05 in all lakes except for the polymictic Forestry Bog (FB) (*P* = 0.10) (Fig. S4A to H).

10.1128/mSphere.00169-17.4 FIG S4  PCoA performed on subsets of the data set. All clustering by layer (A to H) is significant at *P* = <0.005 except the Forestry Bog data, where *P* = 0.101. Given that it is polymictic and shallow and includes only 1 year of sampling, this is not surprising. Clustering is especially prominent in the meromictic lakes. Additionally, each year in each lake has a unique community composition, regardless of layer (I to K). Plots for the epilimnia of lakes shown in Fig. 3 are presented here, using the same analysis as that described in the main text. Only sites with at least 3 years of sampling and no artificial mixing events were analyzed. Download FIG S4, PDF file, 1.4 MB. Copyright © 2017 Linz et al. 2017 Linz et al. This content is distributed under the terms of the Creative Commons Attribution 4.0 International license.

Within layers, mixing regime also explained differences in community composition (Fig. 2; Table S2). Clustering by mixing regime was significant by permutational multivariate analysis of variance (PERMANOVA) in both epilimnion and hypolimnion samples (*r*^2^ = 0.20 and *r*^2^ = 0.22, respectively; *P* = 0.001 in both groups). Site was a strong factor explaining community composition, with significant clustering in epilimnia (*P* = 0.001, *r*^2^ = 0.34) and hypolimnia (*P* = 0.001, *r*^2^ = 0.49) (Table S2). Date and mean water temperature did not describe the observed clustering as well as lake or mixing regime (Fig. S5A to F). Because principal-coordinate analysis can be susceptible to artifacts, we also performed an analysis of beta diversity between sites using a Bray-Curtis dissimilarity distance matrix without ordination; the same results were obtained (Fig. S5G to H). These findings demonstrate that thermal layer, lake, and mixing frequency are associated with changes in bacterial community composition.

10.1128/mSphere.00169-17.5 FIG S5  Alternative colorations of Fig. 2. The ordination displayed in Fig. 2 is presented here with different colorations representing environmental data. Panels A and B show Julian date; no association between community composition and date is observed. Panels C and D color points by mixing regime rather than lake, which was found to be a significant factor explaining community composition in both layers. Panels E and F are colored by the mean water temperature in each layer on the sampling date; results appear associated with mixing regime, especially in hypolimnia. To corroborate the conclusions drawn from ordinations in Fig. 2, we investigated beta diversity between sites using a straightforward distance metric rather than principal-coordinate analysis (G and H). Every sample was compared to every other sample, and the mean pairwise dissimilarity between sites is represented here. Clustering by mixing regime, particularly in hypolimnia, is still observed in this analysis. Download FIG S5, PDF file, 0.2 MB. Copyright © 2017 Linz et al. 2017 Linz et al. This content is distributed under the terms of the Creative Commons Attribution 4.0 International license.

### Variability and dispersion.

While OTU-based community compositions were distinct by layer, lake, and mixing regime, there was still variability over time. We used weighted UniFrac distance to quantify variability in beta diversity between samples within the same site and year. Each year in each lake corresponded to a significantly different community composition, indicating interannual variability in the community composition (Fig. 3A to C; see also Fig. S4I to K and Table S2). As multiple environmental variables changed in each year of sampling, it is not clear which (if any) could explain the observed annual shifts in community composition. We found no evidence of repeating seasonal trends during the stratified summer months in these lakes in time decay plots using weighted UniFrac distance. Likewise, we examined trends in the most abundant individual OTUs and did not observe repeatable annual trends, even when abundances in each year were normalized using *z* scores (Fig. S6).

10.1128/mSphere.00169-17.6 FIG S6  Annual trends in OTUs. We could not identify repeating seasonal trends in OTU abundances. While OTUs tended to show a consistent response to mixing events, their abundance during summer stratification was variable. Example plots showing abundance trends in OTUs over multiple years in the same site are presented here, and readers curious about other OTUs and sites can run the R script “annual_trends_in_OTUs.R” at https://github.com/McMahonLab/North_Temperate_Lakes-Microbial_Observatory for any combination of OTU and location. Download FIG S6, EPS file, 1.6 MB. Copyright © 2017 Linz et al. 2017 Linz et al. This content is distributed under the terms of the Creative Commons Attribution 4.0 International license.

Variability can also be assessed by measuring the beta diversity within a single site. We measured pairwise weighted UniFrac distances between samples in each lake layer (Fig. 3D). This analysis showed that the layers had significantly different levels of pairwise beta diversity within a single site for all lakes except Forestry Bog, as determined using a Wilcoxon signed-rank test with a Bonferroni correction for multiple pairwise comparisons. The mean pairwise UniFrac distance value was lower in the epilimnion than in the hypolimnion in the West and North Sparkling Bogs but was higher in the other significant sites. Performing the same analysis on a single year of data with approximately even numbers of samples from each site showed the same trends. This shows that the amounts of variability in the bacterial community differ by site as well as by year.

### The core community of bog lakes.

Two of the goals of this study were to determine the core bacterial community of bog lakes in general and to determine if the mixing regime affects core community membership. Our previous analyses showed that the community compositions were distinct in each layer and lake (Fig. 2), while marked variability was observed within the same lake and layer (Fig. 3). This prompted us to ask whether we had performed adequate sampling through time and space to fully census the lakes. Still, the rarefaction curves generated for the entire data set and for each layer begin to level off, suggesting that we had indeed sampled the majority of taxa found in our study sites. To identify the taxa that comprise the bog lake core community, we defined the “core” taxa as those present in at least 90% of a group of samples, regardless of abundance in the fully curated data set. Core taxa are reported using OTU number and taxonomic classification in our freshwater-specific database (19). Four OTUs met these criteria for all samples in the full data set: OTU0076 (bacI-A1), OTU0097 (PnecC), OTU0813 (acI-B2), and OTU0678 (LD28). These taxa were therefore also core to both epilimnia and hypolimnia. Additional taxa core to epilimnia included OTU0004 (betI), OTU0184 (acI-B3), OTU0472 (Lhab-A4), and OTU0522 (alfI-A1), while additional hypolimnia core taxa included OTU0042 (Rhodo), OTU0053 (unclassified *Verrucomicrobia*), and OTU0189 (acI-B2).

We performed the same core analysis after combining OTUs assigned to the same tribe (previously defined as sharing ≥97% nucleotide identity in the nearly full-length 16S rRNA gene and according to phylogenetic branch structure [19]) into new groups. This revealed that certain tribes were core to the entire data set or thermal layer even though their member OTUs were specific to certain sites. Notably, some OTUs were endemic in specific lakes, even though the members of their corresponding tribe were found in multiple lakes/layers. OTUs not classified at the tribe level were not included. Results were similar to those observed at the OTU level but yielded more core taxa. Tribes core to all samples included bacI-A1, PnecC, acI-B2, and LD28 but also betIII-A1 and acI-B4. The core tribes in epilimnia were bacI-A1, PnecC, betIII-A1, acI-B3, acI-B2, Lhab-A4, alfI-A1, LD28, and acI-B4, while those in hypolimnia were Rhodo, bacI-A1, PnecC, betIII-A1, acI-B2, and acI-B4. These results show that despite lake-to-lake differences and interannual variability, there are bacterial taxa that are consistently present in bog lakes. We note that tribes correspond very roughly to species-level designations as explained previously (19).

**FIG 4:**
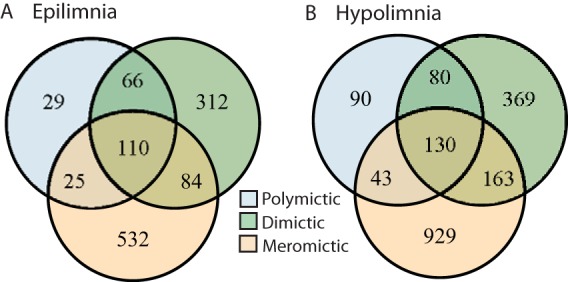
Numbers of unique and shared OTUs by mixing regime. To better understand how shared-community memberships differ by mixing regime, we quantified the numbers of shared and unique OTUs in each category. An OTU needed only to appear in one sample at any abundance to be considered present in a category. We found that in both the epilimnion (A) and hypolimnion (B) layers, meromictic lakes had the highest numbers of unique OTUs and polymictic lakes had the lowest. Meromictic and dimictic lakes shared the most OTUs, while meromictic and polymictic lakes shared the fewest.

Principal-coordinate analysis suggested that samples clustered also by mixing regime (Fig. 2). We thus evaluated Venn diagrams of OTUs shared by, and unique to, each mixing regime to better visualize the overlap in community composition (Fig. 4). In both epilimnia and hypolimnia, meromictic lakes had the highest numbers of unique OTUs whereas polymictic lakes had the lowest, consistent with the differences in richness between lakes (Fig. 1). Shared-community memberships, i.e., the numbers of OTUs present at any abundance in both communities, differed between mixing regimes. Epilimnia (Fig. 4A) and hypolimnia (Fig. 4B) showed similar trends in shared membership: meromictic and dimictic lakes shared the most OTUs, while meromictic and polymictic lakes shared the least.

We next used indicator analysis to identify the taxa unique to each mixing regime. Indicator analysis is a statistical method used to determine if taxa are found significantly more frequently in certain predetermined groups of samples than in others. In this case, the groups were defined by mixing regime, and normalization was applied to account for different numbers of samples in each group. OTUs were grouped at every taxonomic level, and all taxonomic levels were run at once in the indicator analysis to account for differences in the abilities of these levels to serve as indicators (for example, the presence of members of the order *Actinomycetales* is a stronger indicator of polymictic lakes than the presence of members of the phylum *Actinobacteria*). An abundance threshold of 500 reads was imposed on each taxonomic group. The full table of results from the indicator analysis is available as Data Set S1 in the supplemental material, while a few indicator taxa of interest are highlighted here.

10.1128/mSphere.00169-17.10 DATA SET S1  Output of results by indicator analysis. Download DATA SET S1, XLSX file, 0.03 MB. Copyright © 2017 Linz et al. 2017 Linz et al. This content is distributed under the terms of the Creative Commons Attribution 4.0 International license.

Lineage acI is a ubiquitous freshwater group, with specific clades and tribes showing a preference for bog lakes in previous studies (20, 21). Our data set shows a further distinction by mixing regime of acI in epilimnia; acI-A tribes were found predominantly in meromictic lakes, with exception of Phila, which is an indicator of polymictic lakes. Tribes of acI-B, particularly OTUs belonging to acI-B2, were indicators of dimictic lakes. *Methylophilales*, a putative methylotroph, was also an indicator of dimictic lakes, as was the putative sulfate reducing family *Desulfobulbaceae*. The phyla *Planctomyces*, “*Candidatus* Omnitrophica” (formerly OP3), OP8, and *Verrucomicrobia* were found more often in meromictic lakes, as were putative sulfate-reducing taxa belonging to *Syntrophobacterales* and *Desulfobacteraceae*. Indicators of polymictic lakes include ubiquitous freshwater groups such as *Limnohabitans*, *Polynucleobacter* (PnecC), betI-A, and verI-A. Thus, despite the observed variability of and differences between lakes, layers, and years, we detected a core community composed of ubiquitous freshwater bacterial groups. Additionally, we identified indicator taxa endemic to groups of sites defined by mixing frequency.

### Lifestyles of freshwater lineages.

**FIG 5:**
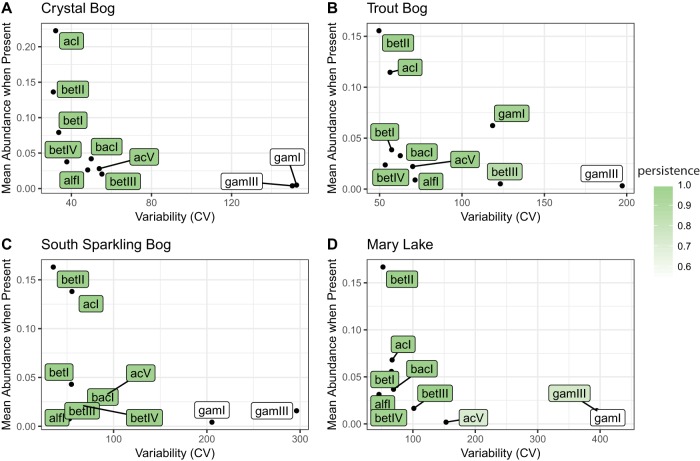
Traits of freshwater lineages. These well-defined freshwater groups showed similar levels of persistence, variance, and abundance in every lake, including Crystal Bog (A), Trout Bog (B), South Sparkling Bog (C), and Mary Lake (D), despite differing abundance patterns. Data from epilimnia with at least 2 years of undisturbed sampling are shown here. Mean abundance values represent the average percentages of reads attributed to each lineage when that lineage was present. Variability was measured as the coefficient of variation (CV). Persistence (shaded color) was defined as the proportion of samples containing each lineage. Additional plots calculated by year can be found in Fig. S7.

Because of the observed variability in bacterial community dynamics, we next asked if individual OTUs showed consistent levels of abundance, persistence, and variability. We defined these metrics as mean abundance (when present), the proportion of samples containing the group of interest, and the coefficient of variation for lineages classified using the freshwater taxonomy, respectively. These metrics have been previously used to categorize OTUs (22, 23). Using only well-defined freshwater groups allowed better taxonomic resolution as we summed the abundances of OTUs by their lineage classification. We note that lineage is very roughly analogous to family in our provisional freshwater taxonomy (19). Lifestyle traits of lineages were consistent both across lakes and across years. Low persistence was associated with high variability, and low variability was associated with high abundance (Fig. 5; see also Fig. S7). We rarely observed “bloomers,” situations where a clade had both high abundance and low persistence; one potential reason for this could be that true “bloomers” drop below the detection limit of our sequencing methods when not abundant. Most freshwater lineages were highly persistent at low abundances with low variability. Lineage gamIII of the gammaproteobacteria, with low persistence, low abundance, and high variability, was an exception. Lineages gamI and verI-A occasionally also exhibited this profile. Lineages betII and acI were highly abundant and persistent with low variability, consistent with their suggested lifestyles as ubiquitous freshwater generalists (12, 21). Even though OTUs did not show the same abundance dynamics each year, they did exhibit patterns that are consistent between years and lakes.

10.1128/mSphere.00169-17.7 FIG S7  Lineage traits by year. Figure 5 demonstrates that lineages show consistent traits in different lakes; this plot shows that those traits are relatively consistent between years in the Trout Bog hypolimnion as well. Download FIG S7, EPS file, 1.6 MB. Copyright © 2017 Linz et al. 2017 Linz et al. This content is distributed under the terms of the Creative Commons Attribution 4.0 International license.

## DISCUSSION

The North Temperate Lakes Microbial Observatory bog data set represents a comprehensive 16S rRNA gene amplicon survey spanning 4 years, eight lakes, and two thermal layers. We hypothesized that alpha and beta diversity would be associated with mixing frequency in bog lakes. Richness and membership in these communities were structured by layer, mixing regime, and lake. However, we found that multiple years of sampling were necessary to census the community of bog lake ecosystems. We identified specific bacterial taxa core to bog lakes, as well as taxa endemic to certain depths or mixing regimes. High levels of variability were detected in this data set; the community composition observed for each lake and each year of sampling was unique. However, freshwater lineages still showed consistent lifestyles, defined by abundance, persistence, and variability, across lakes and years, even though the abundance trends of individual OTUs differed each year. Our results emphasize the importance of the use of multiple sampling events to assess full bacterial community membership and variability in an ecosystem.

The bog lakes in this study have been model systems for freshwater microbial ecology for many years. Early studies used automated ribosomal intergenic spacer analysis (ARISA), a fingerprinting technique for identifying unique bacterial taxa in environmental samples (24). Our research built upon these studies and added information about the taxonomic identities of bacterial groups. For example, persistent and unique bacterial groups were detected in the bog lakes using ARISA (25); we also found persistent groups by the use of 16S rRNA gene amplicon sequencing and could identify them as the ubiquitous freshwater bacteria LD28, acI-B2, PnecC, and bacI-A1. Differences between Crystal Bog, Trout Bog, and Mary Lake (three sites representative of the three mixing regime categories corresponding to polymictic, dimictic, and meromictic conditions) in richness and community membership within 1 year were previously detected (25). Our data supported these results and suggest that these trends are indeed linked with mixing regime, as we included multiple lakes of each type sampled over multiple years in this study.

Our results support previous research on the characteristics of bacterial communities in the epilimnion and hypolimnion and the association of lake mixing frequency with community composition. We confirmed that epilimnion communities tended to be more dispersed than hypolimnion communities, potentially due to increased exposure to climatic events (25). Mixing was disruptive to both epilimnion and hypolimnion communities, selecting for only a few taxa that persist during this disturbance but quickly recovering diversity once stratification was reestablished (11, 26). Our initial inspiration for the collection of this data set was the intermediate-disturbance hypothesis. We hypothesized that water column mixing is a disturbance to bog lake bacterial communities and that lakes with intermediate mixing frequency would have the highest levels of biodiversity. Results of comparisons of levels of richness between lakes with different mixing regimes did not support the intermediate-disturbance hypothesis; rather, the least frequently mixing lakes had the most diverse communities. Richness also correlated positively with lake volume, potentially the result of a positive taxon-area relationship, but analyses of more lakes of similar volumes and of various depths are needed to prove this relationship in our study system (27, 28). As many variables, including mixing frequency and concentrations of nitrogen and dissolved carbon, covary with volume, we cannot determine which of these factors led to the observed differences in diversity between sites based on our data set.

We were not able to detect repeatable annual trends in bog lakes in our multiple years of sampling. While seasonality in marine and river systems has been well established by our colleagues, previous research on seasonality in freshwater lakes has produced inconsistent results (29–32). Distinct, seasonally repeatable community types were identified in alpine lakes, but stratified summer communities were distinct each year (33). Seasonal trends were detected in a time series from Lake Mendota (34) that were similar to those detected in this study, but the summer samples in Lake Mendota were more variable then those collected in other seasons. In the previous ARISA-based research on the bog lakes in our data set, community properties such as richness and rate of change were consistent each year, and the phytoplankton communities were hypothesized to drive seasonal trends in the bacterial communities based on correlation studies (35–37). Synchrony in seasonal trends was observed (36); however, these findings were not reproduced in a second year of sampling for seasonal trends in Crystal Bog and Trout Bog (38). Successional trends were studied in Crystal Bog and Lake Mendota with a relatively small number of samples collected over 2 years, and “dramatic changes” in community composition associated with drops in biodiversity during the summer months were described, while spring, winter, and fall had more stable community composition (35). Because our data set was sparsely represented by seasons other than summer, higher summer variability may explain why we see a different community each year and a lack of seasonal trends in community composition. However, we cannot disprove the influence of seasonality on bacterial community dynamics in temperate freshwater lakes as a general feature. Our results may indeed point to a feature that is unique to darkly stained acidic bog lakes. Even in marine systems, trends in seasonality differ by site and OTU definition, and continued long-term time series sampling is suggested as an approach needed to elucidate these trends and link seasonality in bacterial community composition to biogeochemical cycling (39).

One of the biggest benefits of 16S rRNA gene amplicon sequencing over ARISA is the ability to assign taxonomic classifications to sequences. Tracking bacterial taxa through multiple sites and over multiple years allowed us to detect consistent lifestyle trends, despite a lack of predictability in seasonal trends. Some groups, such as acI (*Actinobacteria*) and betII (*Betaproteobacteria*), were persistent, abundant, and not variable, much like *Pelagibacter ubique* (SAR11) in marine systems. Other freshwater taxa such as gamI and gamIII (both gammaproteobacteria) exhibited a pattern of low persistence, low abundance, and high variability. Unlike in the oceans, where members of taxa such as *Alteromonas* exhibit “bloom and bust” (40), no members of taxa classified within the freshwater taxonomy with high abundance and low persistence or high variability were observed. This suggested either that bog lakes are not conducive to the large blooms of a single population as observed in other freshwater lakes or that taxa with this lifestyle dropped below our detection limit when not blooming.

In addition to a core of persistent taxa found in nearly every sample collected, we also identified taxa endemic to either the epilimnion or hypolimnion and to specific mixing regimes. These endemic taxa likely reflect the physical and/or biogeochemical differences driven by mixing regime. Dimictic and meromictic hypolimnia, which are consistently anoxic, harbor putative sulfur cycling groups not present in polymictic hypolimnia, which are more frequently oxygenated. Members of the acI lineage partition by mixing regime in epilimnia, and the functional traits driving this filtering effect are the subject of active study (20). Interestingly, the meromictic Mary Lake hypolimnion contains several taxa classified into the candidate phylum radiation (41) and a larger proportion of completely unclassified reads than other hypolimnia. This is consistent with the findings of other 16S rRNA gene amplicon sequencing and metagenomics studies of meromictic lakes (42, 43) and suggests that the highly reduced and consistently anoxic conditions in meromictic hypolimnia represent excellent study systems for research on members of the candidate phylum radiation and “microbial dark matter.”

Perhaps the biggest implication of this study is the importance of repeated sampling of microbial ecosystems. A similar data set spanning only a single year would not have captured the full extent of variability observed and therefore would not have detected as many of the taxa belonging to the bog lake community; even our 4 years of weekly sampling during the summer stratified period did not result in level rarefaction curves (see Fig. S7 in the supplemental material). While we found no evidence for seasonal trends or repeated annual trends, it is possible that there are cycles or variables acting on scales longer than the 5 years covered in this data set or that interannual differences are driven by environmental factors that do not occur every year. Unmeasured biotic interactions between bacterial taxa may also contribute to the observed variability. Understanding the factors that contribute to variability in lake communities will lead to improved predictive modeling in freshwater systems, allowing forecasting of bloom events and guiding better management strategies. Additionally, these systems may be ideal for addressing some of the core questions in microbial ecology, such as how community assembly occurs, how interactions between taxa shape community composition, and how resource partitioning drives the lifestyles of bacterial taxa.

To address these issues and more, we continue to collect and sequence samples for the North Temperate Lakes Microbial Observatory, and we are expanding our sequencing repertoire beyond 16S rRNA gene amplicon sequencing. All data we have currently generated can be found in the R package “OTUtable,” which is available on CRAN for installation via the R command line or on our GitHub page. This data set has already been used in a meta-analysis of microbial time series (1). We hope that this data set and its future expansion will be used as a resource for researchers investigating their own questions about how bacterial communities behave on long time scales.

## MATERIALS AND METHODS

### Sample collection.

Water was collected from eight bog lakes during the summers of 2005, 2007, 2008, and 2009, as previously described (25). Briefly, the epilimnion and hypolimnion layers were collected separately using an integrated water column sampler. Dissolved-oxygen levels and temperature were measured at the time of collection using a handheld model 550A meter (YSI, Inc., Yellow Springs, OH). After transport to the laboratory, two biological replicates were taken by filtering approximately 150 ml from each well-mixed sample through 0.22-μl-pore-size polyethersulfone filters (Supor 200; Pall, Port Washington, NY). Filters were stored at −80°C until DNA extraction was performed using a FastDNA Spin Kit for Soil (MP Biomedicals, Santa Ana, CA), with minor modifications (44). The sampling sites are located near Boulder Junction, WI, and were chosen to include lakes that represent the three mixing regimes corresponding to polymictic (multiple mixing events per year), dimictic (two mixing events per year, usually in spring and fall), and meromictic (no recorded mixing events) conditions (Table 1). Trout Bog and Crystal Bog are also primary study sites for the North Temperate Lakes Long Term Ecological Research Program (NTL-LTER), which measures a suite of chemical limnology parameters fortnightly during the open-water season. The NTL-LTER also maintains autonomous sensing buoys on Trout Bog and Crystal Bog, allowing more-refined mixing event detection based on thermistor chain measurements.

**TABLE 1:** Locations and characteristics of study sites^a^

Characteristic	Result(s)							
	Forestry Bog	Crystal Bog	North Sparkling Bog	West Sparkling Bog	Trout Bog	South Sparkling Bog	Hell’s Kitchen	Mary Lake
ID	FB	CB	NS	WS	TB	SS	HK	MA
Depth (m)	2	2.5	4.5	4.6	7	8	19.3	21.5
Surface area (m^2^)	1,300	5,600	4,700	11,900	10,100	4,400	30,000	12,000
Approx vol (m^3^)	867	4,667	7,050	18,247	23,567	11,733	193,000	86,000
Mixing regime	Polymictic	Polymictic	Dimictic	Polymictic	Dimictic	Dimictic	Meromictic	Meromictic
GPS coordinates	46.04777, −89.651248	46.007639, −89.606341	46.004819, −89.705214	46.004633, −89.709082	46.041140, −89.686352	46.041140, −89.709082	46.186674, −89.702510	46.250764, −89.900419
Yr(s) sampled	2007	2007, 2009	2007, 2008, 2009	2007	2005, 2007, 2008, 2009	2007, 2008, 2009	2007	2005, 2007, 2008, 2009
pH	4.97, 4.85	4.49, 4.41	4.69, 4.80	5.22, 5.14	4.60, 4.78	4.46, 4.94		5.81, 5.72
Dissolved inorganic carbon (ppm)	0.94, 1.46	0.69, 1.72	1.12, 2.31	0.76, 1.56	1.73, 4.47	1.97, 6.42	2.91, 9.70	5.54, 12.38
SD	0.28, 1.17	0.15, 0.50	0.23, 0.72	0.17, 0.36	0.66, 54	0.24, 1.56	0.35, 1.03	5.66, 7.69
Dissolved organic carbon (ppm)	10.22, 8.96	15.47, 13.6	10.05, 10.40	7.26, 7.27	19.87, 20.58	12.40, 21.92	7.26, 7.33	20.63, 67.10
SD	0.59, 0.10	4.12, 0.82	1.16, 0.96	0.43, 0.73	2.76, 1.17	0.38, 4.76	1.03, 0.12	1.91, 72.67
Total nitrogen (ppb)		620.57, 846.00	629.09, 809.45		737.71, 1,121.00	813.88, 1,498		1,332.57, 3,652.38
Total phosphorus (ppb)		30.00, 38.86	78.00, 135.45		50.57, 53.25	48.63, 69.14		78.00, 303.50
								
Total dissolved nitrogen (ppb)		1,290.19, 490.13	442.39, 586.56		582.5, 820.21	451.63, 1,179.21		1,024.5, 3,220.14
Total dissolved phosphorus (ppb)		84.25, 14.88	70.22, 22.67		34.5, 31.57	16.25, 18.29		71.13, 228

a The lakes included in this time series are small, humic bog lakes in the boreal region near Minocqua, WI. They range in depth from 2 to 21.5 m and encompass a range of water column mixing frequencies (termed regimes). Dimictic lakes mix twice per year, typically in fall and spring, while polymictic lakes can mix more than twice throughout the spring, summer, and fall. Meromictic lakes have no recorded mixing events. pH was measured in 2007, while nutrient data were determined in 2008 (with the exceptions of FB, WS, and HK, measured in 2007); the two measurements were taken concurrently with the bacterial biomass collection from the same water sample. Where two values are present in a single cell, the first represents the epilimnion value and the second represents the hypolimnion value. GPS, Global Positioning System.

### Sequencing.

A total of 1,510 DNA samples, including 547 biological replicates, were sequenced by the Earth Microbiome Project according to their standard protocols in 2010, using the original V4 primers (FWD, GTGCCAGCMGCCGCGGTAA; REV, GGACTACHVGGGTWTCTAAT) (45). Briefly, the V4 region was amplified and sequenced using Illumina HiSeq, resulting in a total of 77,517,398 sequences with an average length of 150 bp. To reduce the number of erroneous sequences, QIIME’s “deblurring” algorithm for reducing sequence error in Illumina data was applied (46). Based on the sequencing error profile, this algorithm removes reads that are likely to be sequencing errors if those reads are both low in abundance and highly similar to a high-abundance read. Reads occurring fewer than 25 times in the entire data set were removed after deblurring, leaving 9,856 unique sequences. These sequences are considered to represent operational taxonomic units (OTUs).

A total of 570 sequences with long homopolymer runs, ambiguous base calls, or incorrect sequence lengths were found and removed via mothur v1.34.3 (47). Thirty-three chimeras and 340 chloroplast sequences (based on preclustering and classification performed with the Greengenes 16S rRNA gene database, May 2013) (48) were removed. Samples were rarefied to 2,500 reads; samples with fewer than 2,500 reads were omitted, resulting in 1,387 remaining samples. The rarefaction cutoff used was determined based on the results of simulation; a value of 2,500 reads was chosen to maximize the number of samples retained while maintaining sufficient quality for downstream analysis of diversity metrics.

Representative sequences for each OTU were classified in either our curated freshwater database (19) or the Greengenes database based on the output of NCBI-BLAST (blast+ 2.2.3.1) (49). Representative sequences were randomly chosen from each OTU. The program blastn was used to compare representative sequences to full-length sequences in the freshwater database. OTUs matching the freshwater database with a percentage of identity of greater than 98% were classified in that database, and the remaining sequences were classified in the Greengenes database. Both classification steps were performed in mothur using the Wang method (50), and classifications with less than 70% confidence were not included. A detailed workflow for quality control and classification of our sequences is available at https://github.com/McMahonLab/16STaxAss (unpublished data).

### Statistics.

Statistical analysis was performed in R v3.3.2 (51). Significant differences in richness between lakes were tested using a pairwise Wilcoxon signed-rank test with a Bonferroni adjustment in the R package “exactRankTests” (52). Similarities between samples were determined using weighted UniFrac distance, implemented in “phyloseq” (53, 54). Weighted UniFrac distance was chosen because it explained the greatest amount of variation in the first two axes of a principal-coordinate analysis, performed in “vegan” (55). Other metrics tested included unweighted UniFrac distance, Bray-Curtis dissimilarity, and Jaccard similarity; the outputs of all metrics were correlated. Significant clustering by year in PCoA and in dispersion between lakes was tested using PERMANOVA with the function Adonis() in “vegan.” Trimming of rare taxa did not impact the clustering observed in ordinations, such as those present in Fig. 2, even when taxa observed fewer than 1,000 times were removed.

Indicator species analysis was performed using “indicspecies” (56). Only taxa with abundances of at least 500 reads in the entire data set were used for this analysis. The group-normalized coefficient of correlation was chosen for this analysis because it measures both positive and negative habitat preferences and accounts for differences in the numbers of samples from the sites. All taxonomic levels were included in this analysis to determine which level of resolution was the best indicator for each taxonomic group.

Plots were generated using “ggplot2” (57) and “cowplot” (58). “reshape2” (59) was used for data formatting.

### Data availability.

Data and code from this study can be downloaded from the R package “OTUtable” available through the Comprehensive R Archive Network (cran.r-project.org), which can be accessed via the R command line using install.packages(“OTUtable”), and from the McMahon Lab GitHub repository “North_Temperate_Lakes-Microbial_Observatory” (github.com/McMahonLab/North_Temperate_Lakes-Microbial_Observatory). Raw sequence data are available through QIITA (http://qiita.microbio.me) and the European Bioinformatics Institute at accession number ERP016854.
