# Erratum for Linz et al., “Bacterial Community Composition and Dynamics Spanning Five Years in Freshwater Bog Lakes”

**DOI:** 10.1128/mSphere.00296-17

**Published:** 2017-07-19

**Authors:** Alexandra M. Linz, Benjamin C. Crary, Ashley Shade, Sarah Owens, Jack A. Gilbert, Rob Knight, Katherine D. McMahon

**Affiliations:** a Department of Bacteriology, University of Wisconsin—Madison, Madison, Wisconsin, USA; b Department of Microbiology and Molecular Genetics, Michigan State University, East Lansing, Michigan, USA; c Biosciences Division, Argonne National Laboratory, Argonne, Illinois, USA; d Computation Institute, University of Chicago, Chicago, Illinois, USA; e Department of Ecology and Evolution, Department of Surgery, University of Chicago, Chicago, Illinois, USA; f Center for Microbiome Innovation, Jacobs School of Engineering, University of California, San Diego, La Jolla, California, USA; g Department of Pediatrics, University of California, San Diego School of Medicine, La Jolla, California, USA; h Department of Computer Science and Engineering, Jacobs School of Engineering, University of California San Diego, La Jolla, California, USA; i Department of Civil and Environmental Engineering, University of Wisconsin—Madison, Madison, Wisconsin, USA

## ERRATUM

Volume 2, no. 3, e00169-17, 2017, https://doi.org/10.1128/mSphere.00169-17. The GPS coordinates for South Sparkling Bog were reported incorrectly in Table 1; they should instead be 46.003362, −89.705335.

