# Supplementary material for: Bacterial Community Composition and Dynamics Spanning Five Years in Freshwater Bog Lakes
Source: mSphere. 2017 Jun 28;2(3):e00169-17. doi: 10.1128/mSphere.00169-17 (PMC5489657; doi:10.1128/mSphere.00169-17)
Supplement: TABLE S1 [file sph003172312st9.docx]

Epilimnion Hypolimnion

| Site 1 | Site 2 | p-value |  | Site 1 | Site 2 | p-value |
| --- | --- | --- | --- | --- | --- | --- |
| FB | CB | 0.01085 |  | FB | CB | 1.00000 |
| WS | CB | 0.83523 |  | WS | CB | 0.03692 |
| NS | CB | 0.12145 |  | NS | CB | 0.00001 |
| TB | CB | 0.00628 |  | TB | CB | 0.00000 |
| SS | CB | 0.00000 |  | SS | CB | 0.00000 |
| HK | CB | 0.00000 |  | HK | CB | 0.00000 |
| MA | CB | 0.00000 |  | MA | CB | 0.00000 |
| WS | FB | 0.00005 |  | WS | FB | 0.02735 |
| NS | FB | 0.00000 |  | NS | FB | 0.00002 |
| TB | FB | 0.00000 |  | TB | FB | 0.00001 |
| SS | FB | 0.00000 |  | SS | FB | 0.00000 |
| HK | FB | 0.00000 |  | HK | FB | 0.00000 |
| MA | FB | 0.00000 |  | MA | FB | 0.00000 |
| NS | WS | 1.00000 |  | NS | WS | 1.00000 |
| TB | WS | 1.00000 |  | TB | WS | 1.00000 |
| SS | WS | 0.00089 |  | SS | WS | 0.00000 |
| HK | WS | 0.01310 |  | HK | WS | 0.00000 |
| MA | WS | 0.00000 |  | MA | WS | 0.00000 |
| TB | NS | 1.00000 |  | TB | NS | 1.00000 |
| SS | NS | 0.00000 |  | SS | NS | 0.00000 |
| HK | NS | 0.00001 |  | HK | NS | 0.00000 |
| MA | NS | 0.00000 |  | MA | NS | 0.00000 |
| SS | TB | 0.00000 |  | SS | TB | 0.00000 |
| HK | TB | 0.00029 |  | HK | TB | 0.00000 |
| MA | TB | 0.00000 |  | MA | TB | 0.00000 |
| HK | SS | 1.00000 |  | HK | SS | 0.00000 |
| MA | SS | 0.00000 |  | MA | SS | 0.00000 |
| MA | HK | 0.00007 |  | MA | HK | 0.00089 |
