# Supplementary material for: Bacterial Community Composition and Dynamics Spanning Five Years in Freshwater Bog Lakes
Source: mSphere. 2017 Jun 28;2(3):e00169-17. doi: 10.1128/mSphere.00169-17 (PMC5489657; doi:10.1128/mSphere.00169-17)
Supplement: TABLE S2 [file sph003172312st10.docx]

|  |  | Degrees Freedom | Sums of Squares | Mean Squares | F Statistic | | | Partial r^2^ | | | p-value | |
| --- | --- | --- | --- | --- | --- | --- | --- | --- | --- | --- | --- | --- |
| Epilimnion | Lakes | 7 | 2.68 | 0.38 | | 45.85 | | | 0.33 | 0.001*** | | |
|  | Residuals | 663 | 5.53 | 0.01 | |  | | | 0.67 |  | |  |
|  | Total | 670 | 8.20 |  | |  | | | 1.00 |  | |  |
|  | Regime | 2 | 1.52 | 0.76 | | 75.85 | | | 0.19 | 0.001*** | |  |
|  | Residuals | 668 | 6.68 | 0.01 | |  | | | 0.81 |  | |  |
|  | Total | 670 | 8.20 |  | |  | | | 1.00 |  | |  |
| Hypolimnion | Lakes | 7 | 5.25 | 0.75 | | 98.38 | | | 0.50 | 0.001*** | |  |
|  | Residuals | 682 | 5.20 | 0.01 | |  | | | 0.50 |  | |  |
|  | Total | 689 | 10.45 |  | |  | | | 1.00 |  | |  |
|  | Regime | 2 | 3.89 | 1.94 | | 203.49 | | | 0.37 | 0.001*** | |  |
|  | Residuals | 687 | 6.56 | 0.01 | |  | | | 0.63 |  | |  |
|  | Total | 689 | 10.45 |  | |  | | | 1.00 |  | |  |
| Trout Bog | Year | 3 | 0.30 | 0.10 | | 30.25 | | | 0.36 | 0.001*** | |  |
|  | Residuals | 162 | 0.53 | 0.00 | |  | | | 0.64 |  | |  |
|  | Total | 165 | 0.83 |  | |  | | | 1.00 |  | |  |
| South Sparkling Bog | Year | 2 | 0.11 | 0.06 | | 10.57 | | | 0.20 | 0.001*** | |  |
|  | Residuals | 82 | 0.44 | 0.01 | |  | | | 0.80 |  | |  |
|  | Total | 84 | 0.55 |  | |  | | | 1.00 |  | |  |
| Mary Lake | Year | 3 | 0.35 | 0.12 | | | 3.79 | | 0.10 | 0.001*** | |  |
|  | Residuals | 99 | 3.04 | 0.03 | | |  | | 0.90 |  | |  |
|  | Total | 102 | 3.39 |  | | |  | | 1.00 |  | |  |
