# Supplementary figures and images for: Bacterial Community Composition and Dynamics Spanning Five Years in Freshwater Bog Lakes
Source: mSphere. 2017 Jun 28;2(3):e00169-17. doi: 10.1128/mSphere.00169-17 (PMC5489657; doi:10.1128/mSphere.00169-17)

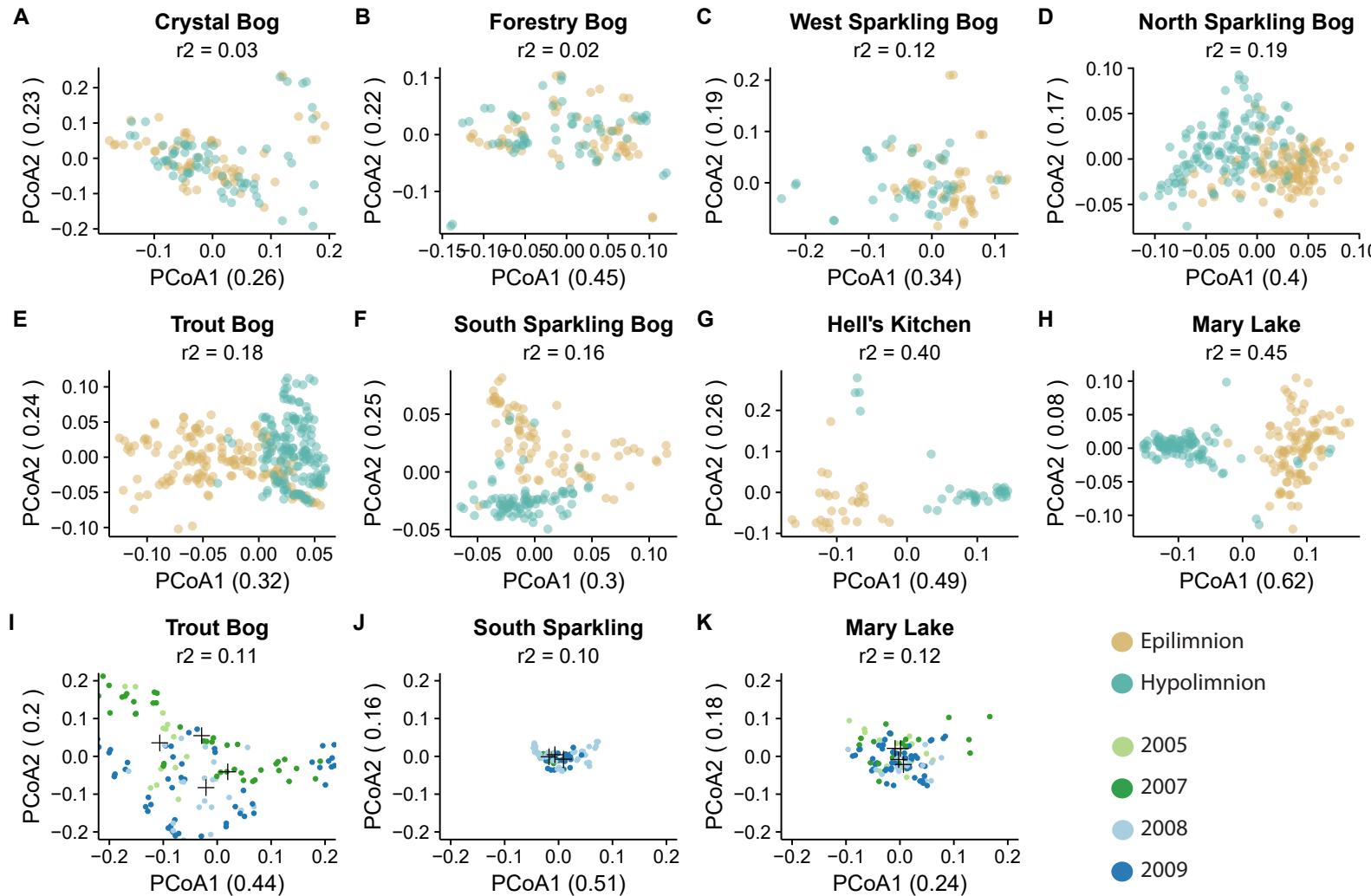

Supplement: FIG S4 [file sph003172312sf5.pdf]

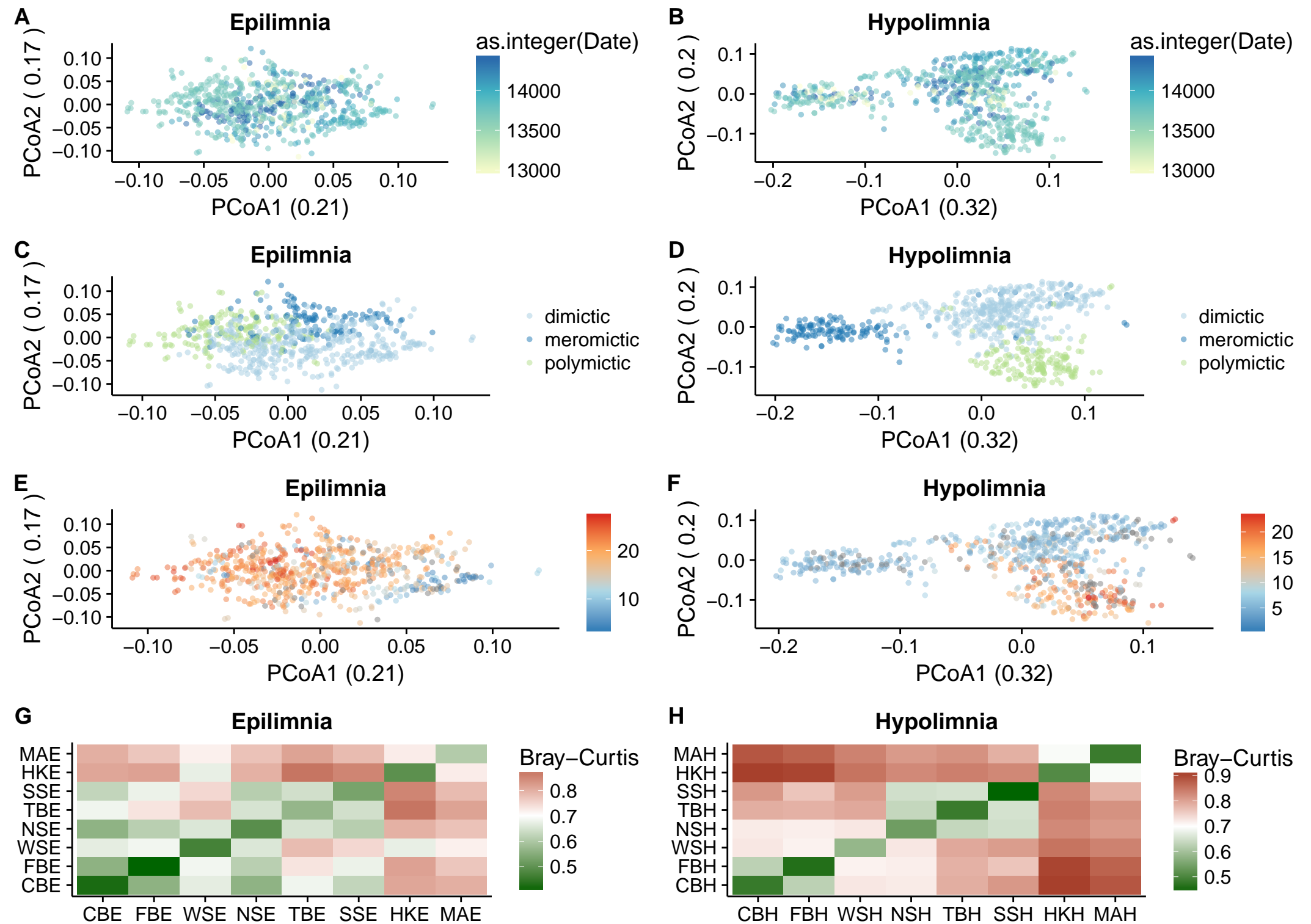

Supplement: FIG S5 [file sph003172312sf6.pdf]
